# Ready Patient One: How to Turn an In-Person Critical Care Simulation Scenario Into an Online Serious Game

**DOI:** 10.7759/cureus.17746

**Published:** 2021-09-05

**Authors:** Colleen M Donovan, Alexandria Cooper, Sarang Kim

**Affiliations:** 1 Department of Emergency Medicine, Rutgers - Robert Wood Johnson Medical School, New Brunswick, USA; 2 Department of Emergency Medicine, Rutgers - New Jersey Medical School, Newark, USA; 3 Department of Medicine, Rutgers - Robert Wood Johnson Medical School, New Brunswick, USA

**Keywords:** healthcare education, covid-19, serious game, escape room, choose your own adventure, cyoa, simulation in medical education

## Abstract

Introduction

Serious gaming has become popular in healthcare education as an engaging way to train learners. When coronavirus disease 2019 (COVID-19) forced the cancellation of in-person simulation sessions, we designed a serious game to deliver content in an interactive format with no out-of-pocket expense. We describe the design process and game reception so that others may replicate it.

Methods

We designed an online game using Choose-Your-Own-Adventure (CYOA) and Escape Room concepts. Using online survey software, we presented an interactive story based on an existing simulation scenario and included interactive puzzles as roadblocks to scenario progression. Each puzzle represented a critical care concept, and many contained hyperlinks to prior basic science lecture material to reinforce learning. A post-game survey assessed students’ experience.

Results

All (N=88) students enrolled in a scheduled simulation session participated in the game, and 75% (66/88) responded to a post-participation survey. All respondents (100%) were able to complete the game. The majority (57.6%) completed the game in 30 minutes to 1 hour. Most students strongly agreed or agreed that the game enhanced their understanding of critical care concepts (93.9-97.0%), and that they were interested in doing more CYOA games (90.9%).

Conclusion

The game was well-received, delivered critical care content, and challenged students to apply basic science principles to medical decision-making from the safety of their own homes. The game was self-guided, requiring minimal active facilitator involvement. We plan to expand the use of the game to other settings and explore its use in formative/summative assessment and remediation.

## Introduction

Simulation is a widely accepted tool for teaching critical care, resuscitation, and medical decision-making. In Spring 2020, the coronavirus disease 2019 (COVID-19) pandemic forced the cancellation of all in-person simulation sessions at our institution. Medical students needed experiential education, “the use of concrete experience (real or simulated) to gain knowledge” [1], more than ever because in less than two years they would join the frontline as first-year residents. Healthcare simulation faculty were challenged to deliver critical care experiences in a safe, remote, and asynchronous manner while maintaining cost and time efficiency. Serious games, where entertainment is secondary to education, offered a unique solution to these conditions [2]. Therefore, we sought to determine whether we could turn an in-person critical care simulation scenario into a minimally-supervised, interactive online game, based on Choose-Your-Own-Adventure (CYOA) and Escape Room concepts. 

The CYOA concept takes its name from a series of interactive fiction gamebooks where readers are presented with choices and their decisions directly affect downstream storytelling [3]. Escape Rooms are games where players are “locked” in a room and must work together to solve inter-related puzzles to escape and win. These games have become popular as entertainment, team-building exercises, and teaching tools. Using these concepts as inspiration, we manipulated free or university-licensed online quiz software (no additional production cost) to create interactive media puzzles that were roadblocks to scenario progression. Each puzzle was designed to teach a critical care concept and may contain links to previously encountered basic science lecture material to reinforce learning. 

A review of current literature reveals that most medically themed CYOA and Escape Room activities successfully apply gamification to student teams who are physically present with each other and facilitators [4-14]. Our game differs from traditional models in that it is completely online, may be played individually or in groups, and requires minimal facilitator support. We found one computer-based CYOA novel and one serious game in the literature that use CYOA and Escape Room concepts similarly to our approach, but they focus on ethics and statistical applications of evidence-based medicine, respectively [15,16]. Our game challenges players to integrate basic science by focusing down to the molecular level and then build their way out to cellular/organ levels to make critical care medical decisions. To our knowledge, similar online games that incorporate this depth and breadth of medical knowledge do not currently exist in the literature. This topic was previously presented as a Simulation Spotlight & Innovations Oral Presentation at the Fourth Annual Tristate Simulation Symposium on September 25th, 2020.

## Materials and methods

Our institution’s IRB approved this study as “Non-Human Subjects Research (including Quality Assurance/Quality Improvement)” because our project was an educational intervention designed to improve upon an existing educational program (New Brunswick Health Sciences IRB 7/2020 Study ID: Pro2020001730).

Audience and survey

Our serious game was used as part of our undergraduate medical education Introduction to Clinical Experience (ICE) week for rising third-year medical students (M3). ICE week occurs at the completion of preclinical years (M1 and M2) as an orientation to clinical rotation expectations. A pre-brief email was sent to the students, describing the serious game concept. Students required a computer with an internet connection and headphones to play the game. An anonymous link was sent to all 88 enrolled M3 students, and they were given one week to complete the game. Two facilitators were available on-call via email during the week to help players troubleshoot the game. 

Using the list of critical actions and puzzle checkpoints (described in detail below), we created a 10-question survey to assess the player's perception of the game’s educational value on a 5-point Likert scale (strongly agree, agree, neutral, disagree, strongly disagree). Participation was voluntary.

Game development 

Our game is composed of three abstract elements: medical scenario, story, and puzzles/gameplay. These elements were made available to players via operational elements, such as survey software and various media. Our design process started with the abstract elements, and then by trial-and-error, we found operational elements to deliver abstract elements. We address these abstract and operational elements below. 

Abstract elements: medical scenario, story & puzzles/gameplay

Medical Scenario

Our process started with choosing the medical content that we wanted to present. The medical scenario was the most straightforward part of game development, as we used the same in-person simulation case that had been used for ICE Week in the past. 

Briefly, the patient is a 50-year-old male with stable angina that devolves into unstable angina and cardiogenic shock. During the in-person simulation, students must achieve certain critical actions (with facilitator guidance) to progress to new phases in the scenario. We took these critical actions directly from the original in-person, manikin-based scenario, slightly augmented them to delve deeper into basic science foundations, and transformed them into checkpoints in the story, as listed in Table 1. 

**Table 1:** In-Person Critical Actions and CYOA Game Checkpoints CYOA: Choose-Your-Own-Adventure; EKGs: electrocardiograms

Critical Actions and Checkpoints
Identify pertinent history elements
Identify/interpret shock physical exam findings
Create a differential diagnosis for life-threatening chest pain
Name different types of shock
Rank shock type likelihood and provide a rationale for ranking
Consider diagnostic testing
Interpret EKGs
Review right ventricular infarct pathophysiology
Choose pathophysiology-based definitive treatment
Review coronary catheterization/angioplasty
Give a report on the patient to the cardiologist

To move forward in the medical scenario/story, players must solve medically themed puzzles related to each critical action/checkpoint (described in more detail below under *Puzzles and Gameplay)*. 

Story

Our next hurdle was the development of characters, a game story, and the fictional world where the medical scenario takes place. In our day-to-day manikin-based simulation sessions, we have a cadre of regular “characters,” especially “Donna” and “Robert,” who are fixtures in many scenarios. Donna is a Socratic character in the role of Charge Nurse. She is a teammate and subtle authority figure, but not supervisory enough to take decision-making away from learners, as an attending physician might. Robert is one of our beloved manikins and plays our patient. The narrative includes the player (the student), Donna (the guide), and Robert (the patient). After a series of mishaps, Donna whisks the player into the hospital and the adventure begins when the player gets nudged into a room where a sick Robert pleads for their help.

**Figure 1:**
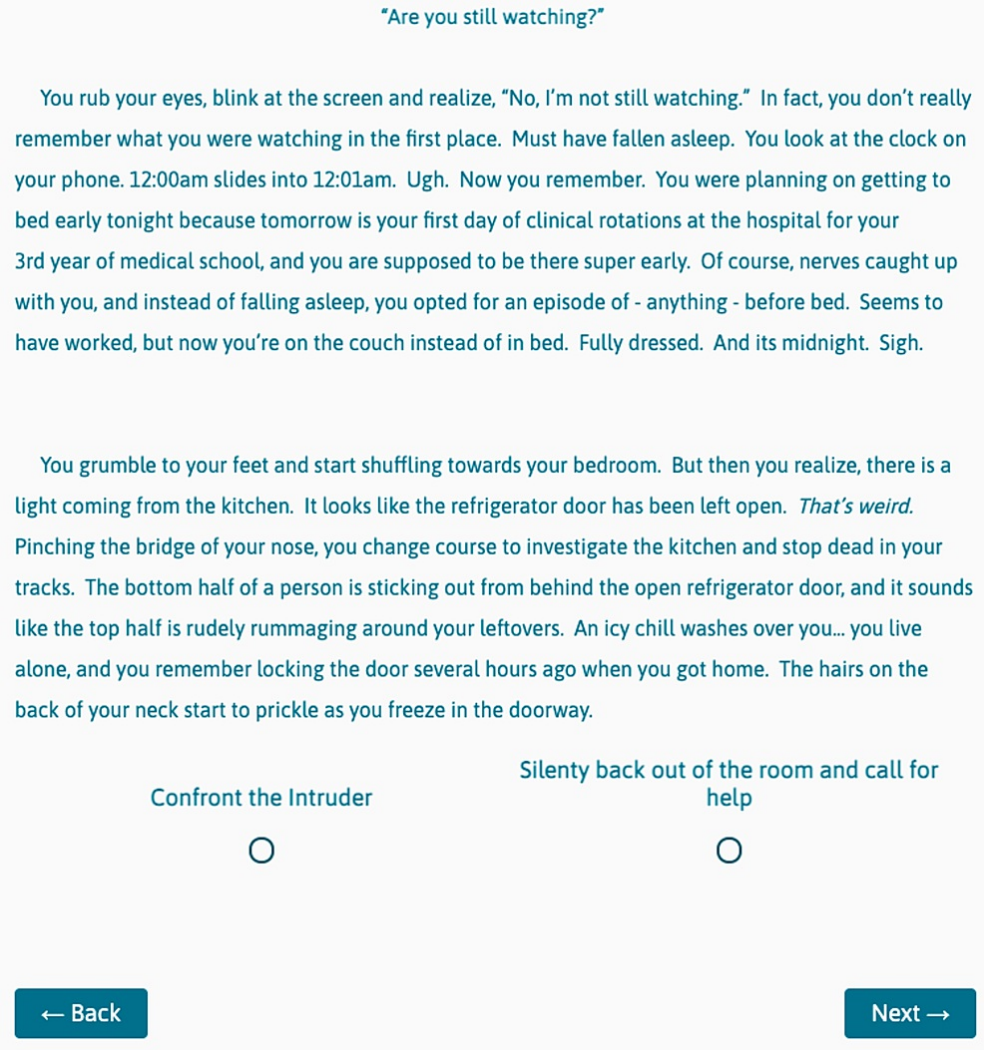
CYOA Game Opening Screen This is the first screen that the player sees. The story progresses based on the choice the player makes at the bottom of this screen.

**Figure 2:**
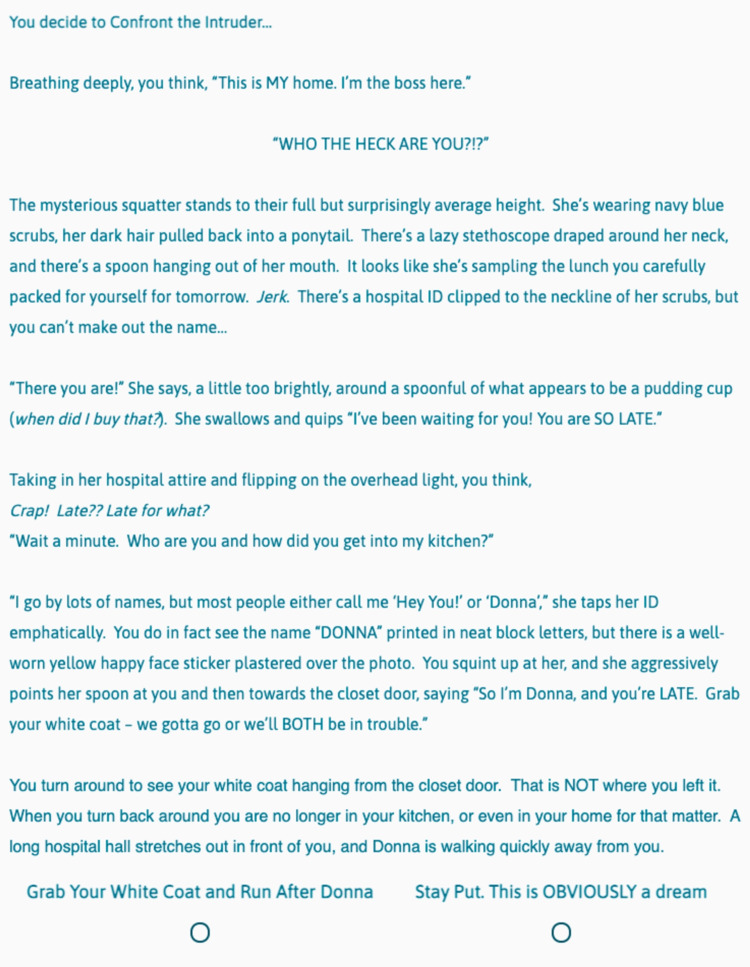
CYOA Game “Confront the Intruder” Screen​ This is the screen that players see if they choose “Confront the Intruder” from Figure 1.​

**Figure 3:**
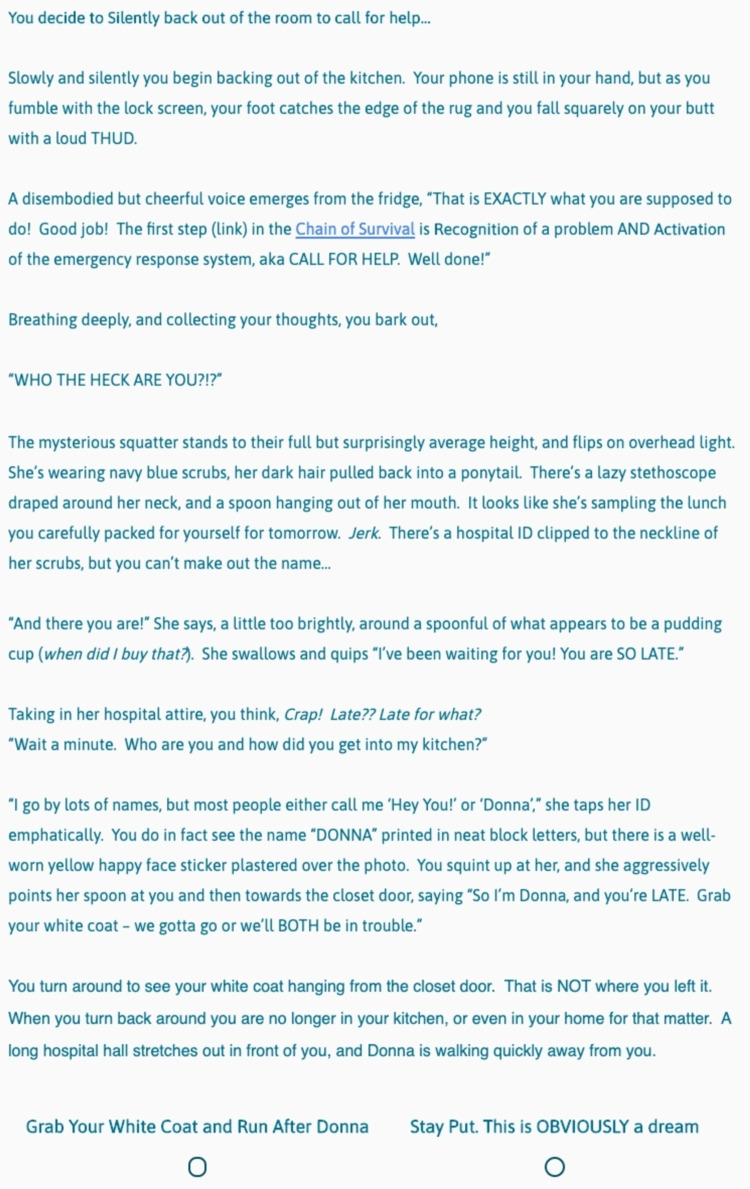
CYOA "Silently back out of the room..." This is the screen that players see if they choose “Silently back out of the room…” from Figure 1.

Our story is a non-linear interactive fiction adventure where player choices affect the flow of the story. Classic CYOA books are examples of interactive fiction and were inspirations for this project. We used a non-linear story format known as a “foldback” story. Foldback stories have branching plot lines but eventually “fold back” to single fixed points. This allows players enough autonomy to explore the game, while also focusing on critical events to move the story forward [17]. For example, from the opening “Are You Still Watching?” Screen (Figure 1), players can choose to “Confront the Intruder” (Figure 2) or “Silently back out of the room and call for help” (Figure 3). 

**Figure 4:**
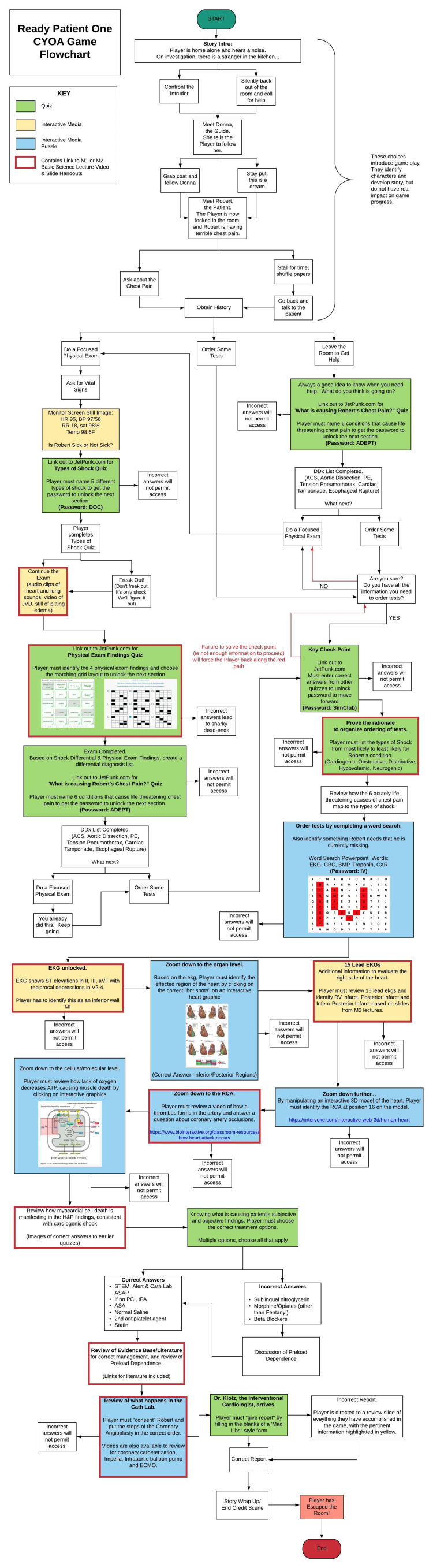
CYOA Flowchart Green boxes indicate associated quizzes. Yellow boxes indicate associated interactive media. Blue boxes indicate associated interactive media puzzles. Red outlined boxes indicate game sections that contain links to basic science lecture videos and handouts.

This first choice (Figure 1) is meant to teach players the mechanics of the game (how to advance or go back, etc.), and therefore does not change the downstream story, as evidenced by foldback or fixed points at the end of both subsequent story choices (Figures 2, 3). Future choices lead to different story paths, but ultimately fold back to critical action puzzles. As the story developed and foldback elements began to take shape, we created a flowchart as a reference (Figure 4). This allowed us to keep track of different plot lines, puzzles, media, and basic science content that players might encounter, depending on their in-game decisions. 

*Puzzles and Gameplay* Once the medical scenario and story were drafted, we turned our attention to puzzles and gameplay. We created 17 individual puzzles that served as roadblocks to continuing the adventure. By manipulating available online survey software, we integrated popular quiz websites, Free Open Access Medical Education (FOAMed), and various other pop culture media to design immersive, interactive, and funny puzzles. These elements were linked to critical actions from the medical scenario (Table 1). Due to the complex nature of some concepts, some critical actions had multiple puzzle elements. Rationales for correct and incorrect answers were provided after puzzle completion with appropriate evidence-based references. Our puzzles fall into three categories: Quizzes (6/17), Interactive Media (5/17), and Interactive Media Puzzles (6/17). 

**Figure 5:**
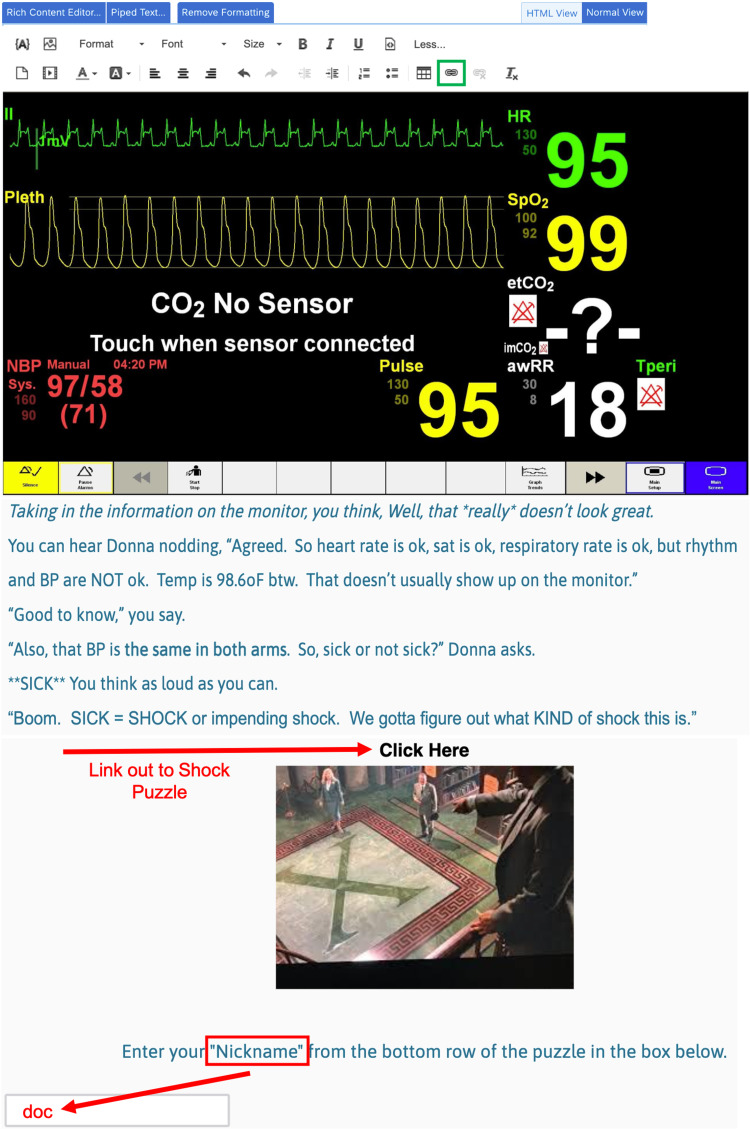
CYOA Shock Quiz Link Out and Password Screen Players encounter this screen before they link to the Shock Puzzle shown in Figure 6.  They return to this screen after completing that puzzle and the password, “doc,” must be entered in the text box in order to progress in the game.  Red arrows show where players must interact with the software. This screen also shows media and links that were added using the Rich Content Editor at the top of the image.  The insert link function is highlighted with a green outline at the top of the figure. This screen correlates to the yellow "Monitor Screen Still Image" box in the flowchart (Figure 4).

**Figure 6:**
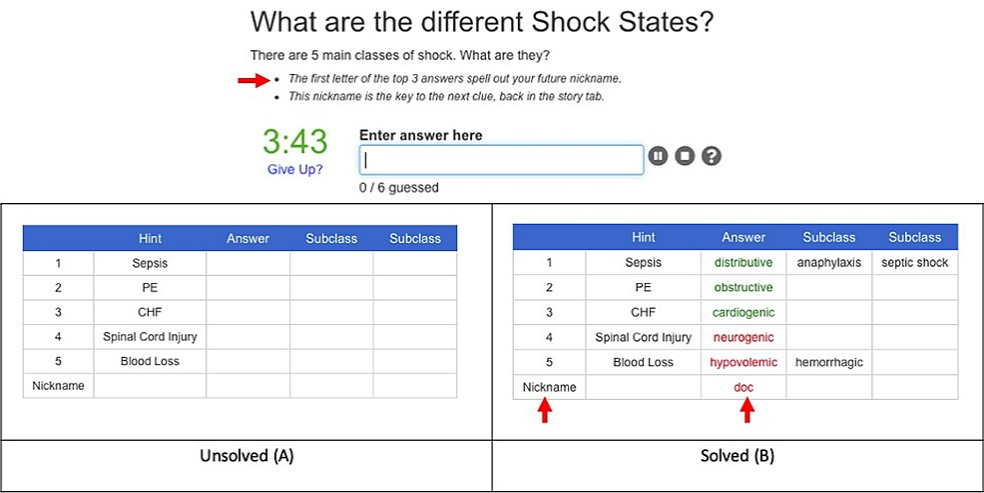
Free-Text Quiz Example: Shock States, Unsolved and Solved This quiz was built on a website called Jetpunk.com.  It requires the player to ​type in the correct answers. The unsolved quiz is shown at the left (A). The solved​ quiz, with three correct answers (green) and three incorrect answers (red), is ​shown on the right (B).  The escape room element is the password, as indicated by the ​clue and the red arrows.  There is a four-minute count-down clock (3:43), and a "Give Up?" link (blue text) in the question stem.  The quiz is solved once all of the correct answers are entered, the timer runs out, or the player selects the "Give Up?" link, allowing eventual game progression even if the player gets stuck on the quiz. Once the player learns the password, they can return ​to Qualtrics and type it into the text box to progress in the game (Figure 5).  This screen correlates to the green "Link out to Jetpunk.com for Types of Shock Quiz" box in the flowchart (Figure 4).

Quizzes were built using free online resources and included multiple-choice questions (MCQs) and free-text quizzes. Free-Text quizzes require players to type in the correct answer instead of choosing from a pre-populated list of multiple-choice answers, therefore challenging the player to supply potential solutions from their knowledge base, as they would at the bedside. We primarily used JetPunk.com for our quizzes due to ease of use, but there are many other platforms with similar options that may be found with a simple internet search. Upon solving the text quiz puzzles, players are provided with a password that accesses the next part of the game (Figures 5, 6). 

**Figure 7:**
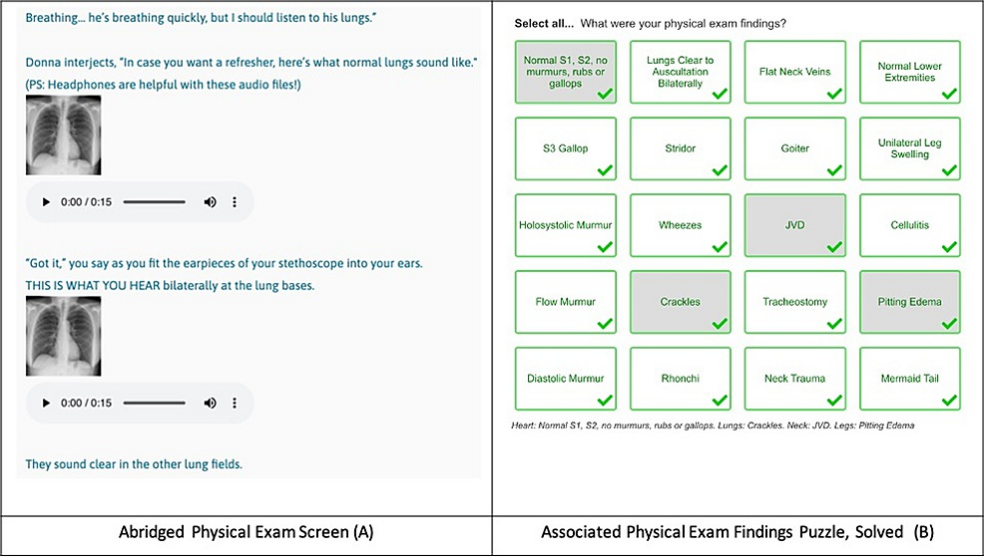
Interactive Media Example (Physical Exam) with Associated Puzzle Players must interact with media by interpreting lung sounds (A) and other audio/visual stimuli in the game. They must then identify their exam findings in the associated puzzle to unlock the password to the next part of the game (B). The Abridged Physical Exam Screen (A) maps to the yellow "Continue the Exam" box and the Associated Physical Exam Findings Puzzle (B) maps to the green "Link out to Jetpunk.com for Physical Exam Findings Quiz" box in the flowchart (Figure 4).

Interactive Media refers to audio/visual stimuli that players must interpret to move forward. We were able to embed audio and visual media into the physical exam and diagnostic checkpoints of our scenario. For example, we were able to provide audio clips of heart and lung sounds (recorded using a digital stethoscope). The associated critical action puzzle requires players to identify the sounds correctly in order to move forward (Figure 7).

**Figure 8:**
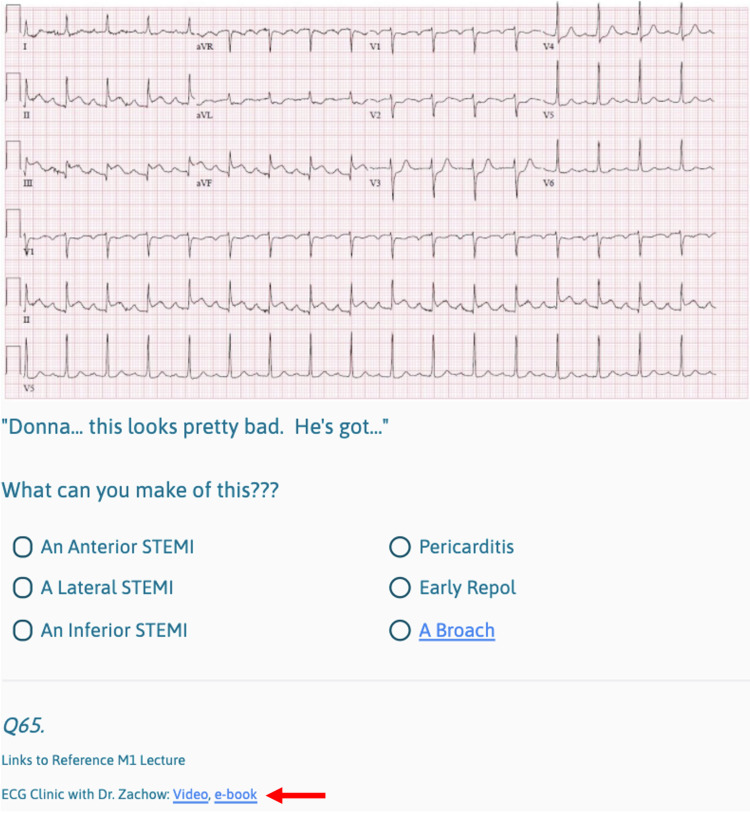
Interactive Media Example (EKG Interpretation) with Basic Science Reference Link Players must correctly interpret this EKG in order to progress in the game. Links to associated basic science lectures and resources are embedded at the bottom of the page (red arrow). This screen maps to the "EKG unlocked" yellow box with a red outline in the flowchart (Figure 4).

Another Interactive Media example is Robert’s EKG, which must be correctly interpreted for players to progress (Figure 8).

**Figure 9:**
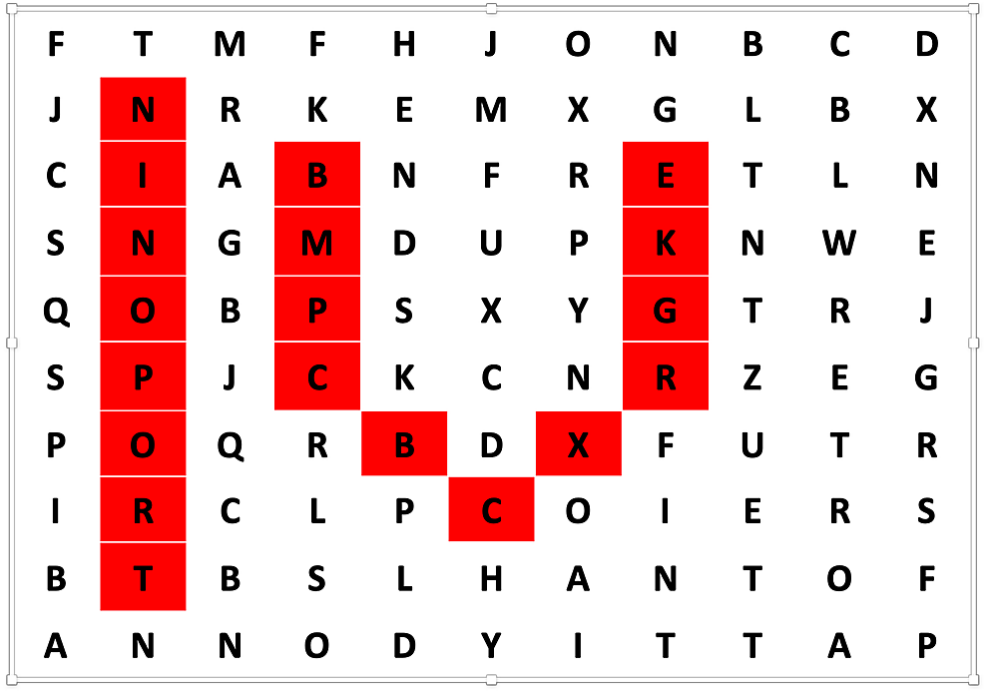
Interactive Media Puzzle Example (Diagnostics Word Search, Solved) Players must solve this word search by finding the appropriate diagnostic tests (e.g., TROPONIN, CBC, BMP, EKG, CXR in the given figure). The highlighted answers reveal the password to the next game section, “IV.” This screen maps to the blue "Order tests by completing a word search" box in the flowchart (Figure 4).

Interactive Media Puzzles require players to physically manipulate an image or audio/video clip to move forward. For example, in choosing diagnostic testing, players must find the names of the correct diagnostic tests in a word search. The highlighted terms spell out the password to the next phase of the game (Figure 9). 

Another example includes a hyperlink to an online 3D heart model that has numbered anatomic landmarks. Players must manipulate the model in three dimensions to identify the source of Robert’s myocardial infarction. Players must make note of the blood vessel and its associated number, and in doing so, review cardiac anatomy (blue "Zoom down further" box in Figure 4). 

Operational elements: platform, basic science references, timeframe

Platform

Choosing an overarching platform to combine the story, medical scenario and puzzles was a challenge. Online survey software was a logical choice for the CYOA concept, as surveys require participants to choose from a set list of options, while streamlining the design process for the surveyor. Online form/survey software is already being used by some educators to build CYOA projects with their students [18,19]. We tried several available online survey platforms (Table 2) but ultimately decided on Qualtrics (Qualtrics, Provo, UT, USA), as it is licensed by our institution, has broad question-type variety, and has relatively user-friendly logic tools. While we chose to use our university-licensed survey platform, it must be noted that there are several free survey software platforms that offer similar, albeit limited, functions. Creative, motivated educators could apply our structure even to the most basic of plans or totally free, open-source software [20]. 

**Table 2:** CYOA Most Used Survey Design Tools and Examples List of encountered game design problems, the functions that address these problems, the names of these functions in common survey software (Qualtrics, Google Forms, Microsoft Forms, Survey Monkey), and the figures that show a solution example.

Problem	Function	Qualtrics	Google Forms	Microsoft Forms	SurveyMonkey	Example
Need to create separate pages so that players can’t read ahead and skip sections of the game.	Create a new page	Add Block	Add Section	Add Section	Add New Page	Figures 1-3
Need to prevent players from moving to the next page without choosing a path.	Roadblock to force a choice	Force Response	Required Toggle	Required Toggle	Options: Require	Figure 10
Need to create a path from a given choice to the next page in that storyline.	Conditional or If/Then Formatting	Display Logic	“Go to section based on answer”	Add Branching	Logic	Figure 12
Need to add design elements, such as different color text, fonts, interesting images, funny memes or other media to make the game fun.	Design editing	Rich Content Editor	Add Image, Add Video	Insert Media	Advanced Editor	Figure 5
For puzzles where the solution is the password to the next section, need a way to prevent progress unless the correct password is entered.	Validation	Custom Validation	Response Validation	Restrictions: Custom	Options: Validate	Figure 11

Through trial-and-error, we were able to create logic paths and custom validation for each question that permitted forward flow, created a roadblock, or re-routed players to another section depending on players’ choices (Figure 4). 

**Figure 10:**
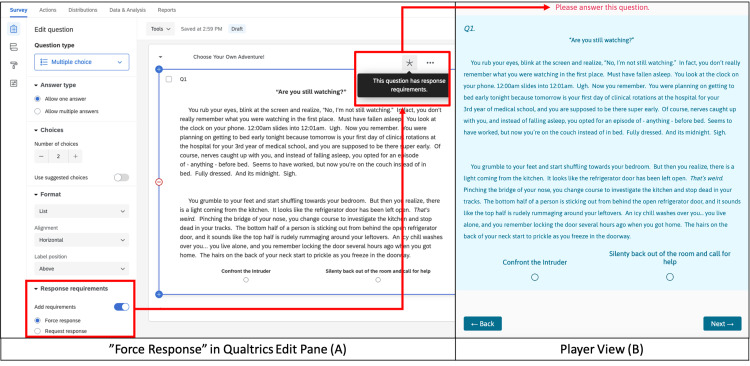
Force Response Example This figure shows "Force Response” in the Qualtrics edit pane (A) for the opening screen (Figure 1), and subsequent player view if no selection is made (B). It is important to select “Force Response” for every section/block of the story in the edit pane to prevent the players from reading ahead or out of order (A). If no selection is made and the player tries to advance, “Force Response” causes an error message to be displayed, “Please answer this question," in the player view (B).

**Figure 11:**
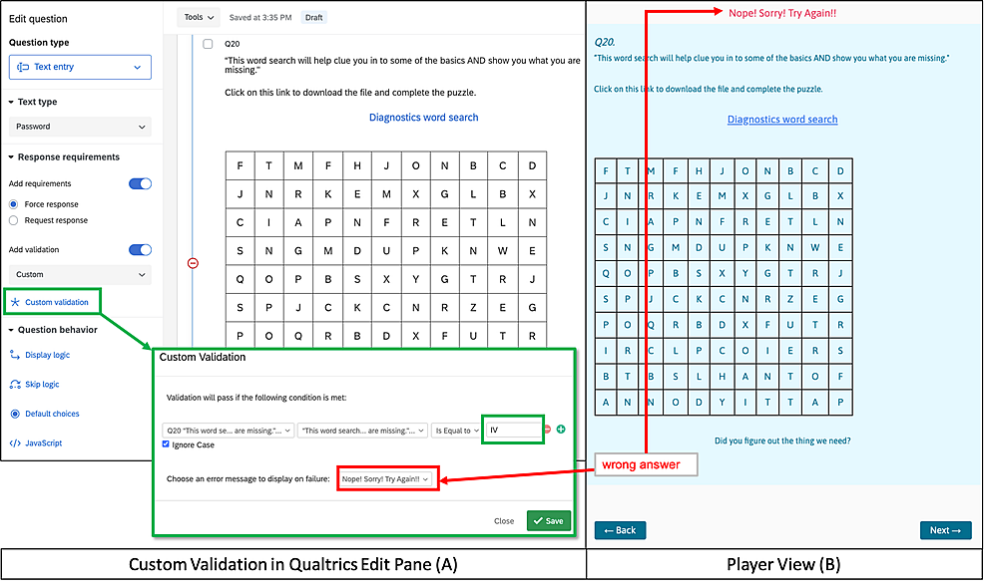
Custom Validation Example Custom validation was used in multiple puzzles, such as the diagnostics word search (Figure 9), to prevent players from skipping ahead. The solution to the word search puzzle is “IV.” If players type in “IV,” they progress to the next page because our custom validation rule states that the answer must be "Equal to… IV” (green boxes/arrows) in the edit pane (A). If any other answer is entered in the text box (i.e. "wrong answer"), an error message, “Nope! Sorry! Try Again!!” appears (red boxes/arrows) in the player view (B). Additional hints to correct answers were included in the puzzles to prevent students from getting "trapped"/unable to progress.

Survey software programs have multiple built-in tools that allow the surveyor to manipulate how their information is viewed by the participant. The tools we used most are summarized in Table 2 with illustrated examples in referenced figures. By manipulating question types, forced responses, and validation rules, we were able to block players’ forward progress unless they correctly solved associated critical action puzzles (Figures 10, 11). 

**Figure 12:**
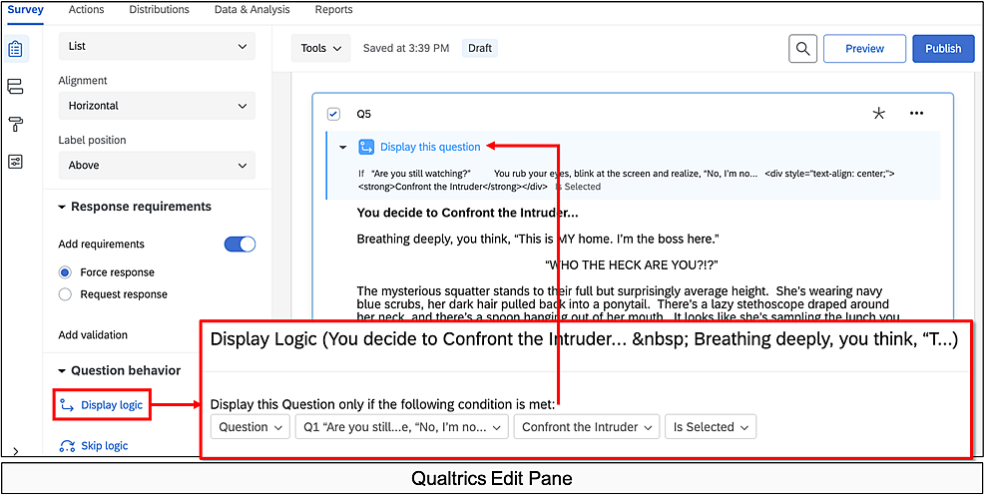
Display Logic Example Logic tools allow for specific information to be displayed if a specific condition is met.  For example, in the opening “Are you still watching?” screen (Figure 1), players can choose to “Confront the Intruder” or “Silently back out of the room and call for help.” Two different subsequent storylines were written, one for each choice, and each placed in a new block or section (Figures 2, 3). Choosing to add Display Logic in this edit pane tells the software to display the “Confront the Intruder” story page ONLY IF the “Confront the Intruder” button is selected in the previous screen (Figure 1). The “Silently back out of the room and call for help” text is similarly displayed if that choice is selected.  In stories with multiple choices or branch points, the logic can get very confusing and complicated. Creating a flowchart (Figure 4) is helpful in keeping the logic organized and preventing players from getting “lost” or trapped in dead-ends.

Display logic was frequently used to route incorrect attempts to humorous error messages and internet memes. Supplementary plotlines with clues or leading questions (provided by Donna) were included to aid players if they supplied incorrect answers or got stuck on a puzzle, therefore preventing players from getting trapped at any given point in the game (Figure 12). 

As noted above, some puzzles, quizzes, and media were created or located on external websites. In order to direct players to the appropriate roadblock puzzles, links to these quizzes and media were embedded into the Qualtrics screens (Figure 5). In order to prevent navigation away from the main Qualtrics game screen, we used the Rich Content Editor (also seen in Figure 5) which allows for changes of design, including font, color, the addition of picture or video files, and insertion of hyperlinks. Using the link function, we entered the quiz web address, and using the target function, we chose “New Window,” which opens quizzes and other media in a new tab without disturbing the main Qualtrics game screen.

Upon completion of the game platform in Qualtrics, an anonymous link was sent to the students/players. We chose to send an anonymous link to encourage the students to explore the educational activity without worrying about faculty being able to view their individual performance.

Basic Science References

Early in development, we reached out to M1/M2 course directors responsible for the basic science topics that were pertinent to our scenario. They provided lecture slides and recordings, and some of that content was included in the game. At our institution, basic science lectures are recorded and saved to our online academic management platform. Students can watch the videos and download slide handouts on demand. The academic management platform is password protected and only available to students and faculty at our institution. We embedded direct links to the basic science lecture videos and handouts for ease of cross-reference for players (Figure 8). On our flowchart (Figure 4), a red outline denotes game areas where direct links to basic science content are available to players. 

Timeframe

Game development took approximately six weeks. The story and medical scenario took approximately two weeks to draft. Appraising and choosing an overarching online survey platform, building and testing puzzles, experimenting with free online puzzle software, and sourcing and embedding relevant media took three weeks. Finally, a small group of alpha and beta testers (faculty and medical students/residents, respectively) was recruited to play the game, which took an additional week.

## Results

The post-game survey was sent to all 88 enrolled M3 students. We had a 75% response rate (66/88). All respondents (100%) were able to complete the game. The majority of students (57.6%, 38/66) completed the game in 30 minutes to 1 hour, and many others completed the game in 1 to 2 hours (33.3%, 22/66). Most students strongly agreed or agreed that the game enhanced their understanding of critical care concepts (93.9-97.0%), that they enjoyed the game (92.4%), and that they were interested in doing more CYOA games (90.9%). All free-text comments about the game were positive, and representative comments are included in Table 3. 

**Table 3:** Post-Game Survey Responses Summary of the post-game survey responses, including representative comments.

Question	Strongly Agree N (%)	Agree N (%)	Neutral N (%)	Disagree N (%)	Strongly Disagree N (%)
I feel I better remember the 5 different types of shock.	38 (57.6%)	24 (36.4%)	2 (3.0%)	2 (3.0%)	0
I feel I better understand the "cannot miss" differential diagnoses for chest pain.	48 (72.7%)	14 (21.2%)	3 (4.6%)	1 (1.5%)	0
I feel I better understand the basic science and diagnosis of ACS and Inferior Wall MIs.	38 (57.6%)	25 (37.9%)	1 (1.5%)	2 (3.0%)	0
I feel I better understand the acute management and treatment options for ACS and Inferior Wall MIs.	41 (62.1%)	23 (34.9%)	1 (1.5%)	1 (1.5%)	0
I enjoyed the CYOA Game.	40 (60.6%)	21 (31.8%)	2 (3.0%)	3 (4.6%)	0
I would be interested in doing more CYOA Games based on other medical topics.	37 (56.1%)	23 (34.9%)	3 (4.6%)	3 (4.5%)	0
Representative comments	“I thought that the game… [was] extremely well put together and well done. Easily the best remote learning opportunity I've had since COVID-19 started and made really effective use of the online tools available to us. It was very clear that [faculty] not only had knowledge of how to use the remote learning tools, but [were] also interested in utilizing them to their best capacity to make the experience fun and engaging.”				
	“The CYOA game was fantastic. This was a really excellent use of my time and actively kept me engaged. Not only did it get me to think about tricky topics, but I also found that I remembered more than I thought I did… I really do hope that this type of exercise continues in the future because it was one of the most engaging sessions!”				
	“I just wanted to say that it was very clear [faculty] put in a lot of time and effort into that and it was not unnoticed. I have always dreaded doing other simulations or modules, but I actually had fun during this one! Thank you for making this experience as enjoyable as it could have been given the COVID situation!”				

Of the 88 participating students, only six reached out to the facilitators during the week of the game; all concerns were related to minor technical issues that were promptly resolved.

## Discussion

Serious gaming has become increasingly popular in healthcare education as an engaging way to train learners. From 2007-2014, serious medically themed games increased from two games across two genres to 42 games across eight genres [21]. Compared to traditional in-person simulation, computer-based games may be less resource-intensive and easier to implement in the setting of busy learner and faculty schedules [22]. 

While serious games are attractive to learners because they are fun, the conceptual framework and educational benefit are explained by adult educational theories such as cognitive constructivism. Players are subjected to cognitive conflict within the safe confines of the game and forced to “construct” conceptual scaffolds by building new knowledge on what was previously known [23,24]. When we expose players to the game (concrete experience), they enter Kolb’s experiential learning cycle. By observing and reflecting on game rules and parameters, the player forms and tests new concepts and solutions in order to complete the adventure and win the game. The experiences and memories created from a simulation game can then be applied to future real-life scenarios, and Kolb’s cycle continues [4,24,25].

Storytelling is an important part of game and simulation design because it creates an immersive environment for players. “Pretending” and “suspension of disbelief” are core concepts in both game design and educational simulation [2,26,27]. A compelling story helps players to suspend disbelief and promotes “emotional satisfaction” by giving players a “dramatically meaningful, rather than abstract, goal” [26]. Including pop culture references, such as the common video streaming service prompt of “Are you still watching?” is one way of capturing the player’s attention. 

In addition to the environment, it is important that students feel a connection with simulated patients and team members. In order for students to buy into the scenario, they must believe that these characters could exist in the real world, and that they could care about these characters. This emotional connection aids in creating long-term memories that are then applied by the adult learner in cognitive constructivism and Kolb’s cycle [4,23,28]. Storytelling thus provides emotional context to the medical scenario and embedded puzzles, weaving these elements together to support learner engagement and material retention. 

Non-Player Characters (NPCs), such as Donna and Robert, help foster an immersive environment and, with careful character development, promote the emotional connection that assists the learner in achieving educational goals [29]. Our NPCs also allowed our game to be self (or character) guided. Students, therefore, required minimal facilitator involvement, subsequently freeing up faculty to focus on clinical duties during the pandemic.

Our findings show that with careful attention to the above-mentioned abstract and operational elements, we successfully converted a simulation scenario into a serious game at a minimal cost. The post-game feedback was overwhelmingly positive, and some of our students found the game to be one of the best, most engaging remote exercises to date.

While online serious gaming is an innovative solution to many problems, it is not a simple task. Our main challenge was the amount of time it took to develop this exercise, taking six weeks of steady work. Despite the significant upfront investment, we now have an exercise that is self-facilitated, easy to implement, and modifiable to suit different settings, specialties, and levels of learner with minimal additional work.

## Conclusions

Our study demonstrates that it is possible to design and implement a minimal cost, well-received serious game based on an in-person simulation. Our game challenges students to create critical care differential diagnoses from history and physical exam components, to choose and interpret diagnostic testing, and to apply basic science principles to medical decision-making, all from the safety of their own homes with minimal facilitator involvement. 

Many important lessons were learned in the development of our game. One of the most helpful tools in our process was the flowchart (Figure 4), as this was our master game map. In story development and writing, we found it helpful to write out one entire block choice before tackling the other options. For example, we wrote the entire "Confront the Intruder" block seen in Figure 2 first. Then, because this was a fold-back point early in the game, we were able to write two new paragraphs for another option, the "Silently back out of the room..." block (Figure 3). The rest of the Figure 3 block is essentially identical to Figure 2, except for minor details, such as who turns on the light. This helped prevent the story from diverging too much and kept our players on track. When considering branching points and display logic, we found it very helpful to include the player's decision at the top of the subsequent block. For example, when the player chooses to "Confront the Intruder" in Figure 1, our display logic moves them automatically to the screen seen in Figure 2. Having the text "You decide to confront the intruder..." at the top of the subsequent block reminds the player where they are in the story (a concept borrowed directly from the classic Choose Your Own Adventure Books) and makes it easier to insert the display logic, as the name of the route is immediately at the top of the block. Finally, in quiz and puzzle design, it is important for the designers to have fun with the puzzles. If the designers don't enjoy the games, it is unlikely that the players will enjoy them. When the design process became frustrating or tiring, we made ourselves take a break and ask colleagues for help or inspiration. This allowed us to come back to the design with fresh and fun ideas that everyone enjoyed.

In the future, we will provide the current game to other learners, such as M4s and residents to review cardiogenic shock. We plan to explore its use as a formative exercise, summative assessment, and remediation exercise, to enhance clinical reasoning or application of previously taught concepts to the clinical setting. Further evaluation is needed to test post-game retention of material and compare the achievement of learning objectives with in-person simulation versus serious gameplay. Finally, as COVID-era restrictions continue, we are working to create more adventures that follow this model and hope that others will be able to apply this design process to their own games. 
